# Comparative study of binding pocket structure and dynamics in cardiac and skeletal myosin

**DOI:** 10.1016/j.bpj.2022.11.2942

**Published:** 2022-11-29

**Authors:** Anna Katarina Antonovic, Julien Ochala, Arianna Fornili

**Affiliations:** 1 School of Physical and Chemical Sciences, Queen Mary University of London, London E1 4NS, United Kingdom; 2 Department of Biomedical Sciences, University of Copenhagen, København N 2200, Denmark; 3 Centre of Human and Applied Physiological Sciences, King’s College London, London SE1 9RT, United Kingdom

## Abstract

The development of small molecule myosin modulators has seen an increased effort in recent years due to their possible use in the treatment of cardiac and skeletal myopathies. Omecamtiv mecarbil (OM) is the first-in-class cardiac myotrope and the first to enter clinical trials. Its selectivity toward slow/beta-cardiac myosin lies at the heart of its function; however, little is known about the underlying reasons for selectivity to this isoform as opposed to other closely related ones such as fast-type skeletal myosins. In this work, we compared the structure and dynamics of the OM binding site in cardiac and in fasttype IIa skeletal myosin to identify possible reasons for OM selectivity. We found that the different shape, size, and composition of the binding pocket in skeletal myosin directly affects the binding mode and related affinity of OM, which is potentially a result of weaker interactions and less optimal molecular recognition. Moreover, we identified a side pocket adjacent to the OM binding site that shows increased accessibility in skeletal myosin compared with the cardiac isoform. These findings could pave the way to the development of skeletal-selective compounds that can target this region of the protein and potentially be used to treat congenital myopathies where muscle weakness is related to myosin loss of function.

## Significance

In this work, we use molecular modeling to give new insight into the molecular basis of selectivity of omecamtiv mecarbil (OM), the first myosin modulator to enter clinical trials. By comparing the structure and dynamics of the region corresponding to the OM binding site in cardiac and in skeletal myosin, we found important differences in the shape and size of the binding pocket in the two isoforms, which could be exploited for the rational design of novel skeletal-selective compounds. We also found that considering protein dynamics is essential to observe binding affinities consistent with OM selectivity for the cardiac isoform.

## Introduction

Direct targeting and modulation of sarcomeric proteins, in particular myosin, has recently shown promise as a pharmacological treatment for several diseases. In fact, mutations in genes encoding sarcomeric proteins are linked to functional and structural alterations at the myofilament level, which can cause congenital cardiac and skeletal myopathies (1). This has led to the development of different myosin-targeting drugs that have reached the clinical trials stage, including the myosin activators omecamtiv mecarbil (OM) (2,3) and danicamtiv (4), and the inhibitors mavacamten (5) and aficamten (6).

An important property of these myosin modulators is their selectivity toward the cardiac isoform of myosin, which makes them ideal candidates for the treatment of cardiovascular disease. Selectivity of a drug is important for achieving desired pharmacological effects and avoiding off-target toxicity, but determining why a drug is selective is often difficult, especially when there is high sequence and structural conservation between the target protein and other related homologous proteins.

OM was the first of these drugs to be developed and to enter clinical trials (first-in-class cardiac myotrope). Its effect on myosin function and structure has been extensively studied (2,7,8,9,10,11,12,13,14,15,16,17,18,19), but the reasons for its selectivity are not yet fully understood. The compound binds to an allosteric site where it interacts with elements that stabilize the pre-power stroke (PPS) state of myosin and control the lever arm swing (7,8). The availability of structural information on its binding site makes it an ideal candidate to investigate the molecular basis of its selectivity.

In this study, we used molecular modeling and simulation to characterize and compare the structure and dynamics of the OM binding region in the motor domain of the human slow/beta-cardiac myosin heavy chain (MYH7 or cardiac myosin for simplicity in the following) and of the human fast type IIa myosin heavy chain (MYH2 or skeletal myosin for simplicity), one of the most abundant isoforms in skeletal muscle. We identified significant differences in the volume and shape of the target binding site, which provide new insight into the reasons for OM selectivity and at the same time give important structural information for the future targeting of skeletal myosin isoforms in this region.

## Results

### Comparison of the OM binding region in cardiac and skeletal myosin

**Figure 1:**
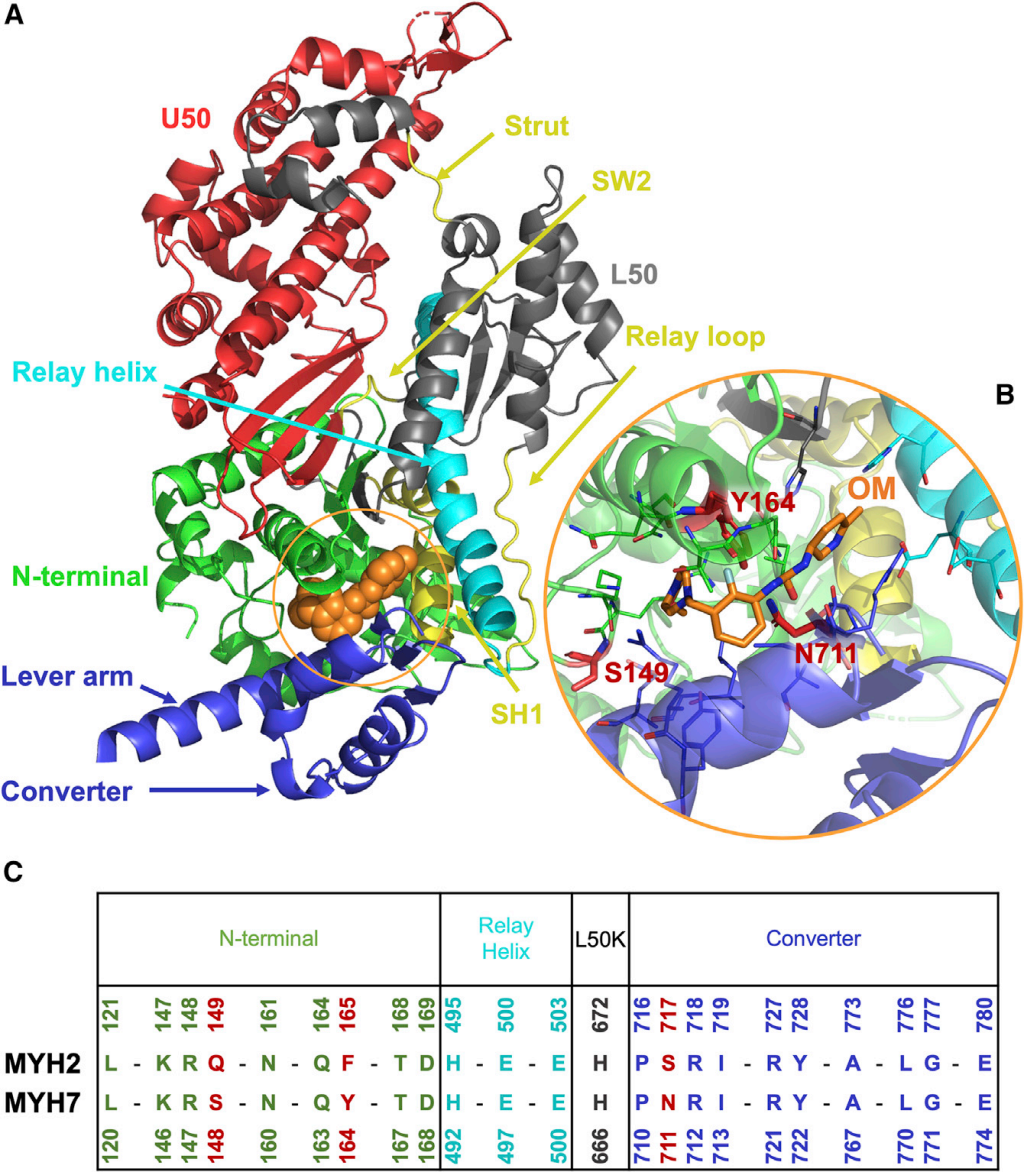
OM binding site. (*A*) Cartoon representation of the x-ray structure of cardiac myosin bound to OM (orange spheres) (PDB: 5N69). The different subdomains are highlighted using different colors. OM is shown as orange spheres. (*B*) Close-up view of the OM binding site. (*C*) Alignment of the human MYH2 (skeletal) and MYH7 (cardiac) sequences in the OM binding region. The residues that differ in the two sequences are highlighted in red. To see this figure in color, go online.

The myosin binding pocket targeted by OM is located at the interface between different key regions in the protein, namely the N-terminal domain, the relay helix, the converter, and the lever arm (Fig. 1). Comparing the MYH2 (human skeletal) and MYH7 (human cardiac) sequences in the binding region shows differences at three positions (red in Fig. 1 *B* and *C*). In particular, residues Y164_c_ and N711_c_, which face each other in the cardiac binding site (Fig. 1 *B*), are replaced by the shorter F165_s_ and S717_s_ in the skeletal sequence (Fig. 1 *C*). Here and in the following, subscripts s and c in the residue names indicate whether the numbering is from the MYH2 or MYH7 sequences, respectively (Table S1).

To investigate the effect of these differences on the binding properties of this site in the two isoforms, we generated models of human MYH2 in the PPS conformation using homology modeling and molecular dynamics (MD) simulations (see methods for more detail). Two popular homology modeling tools, Modeller (M1 model) and Swiss-Model (M2 model), were used to assess the dependence of the results on the specific modeling method. The two models were found to have comparable quality as measured by QMEAN and MolProbity scores (Table S2), with the M2 model having a higher similarity to the cardiac template (Table S3). Both models led to stable MD simulations, with the C_α_ root mean-square deviation (RMSD) from the initial structure quickly reaching a plateau at ∼ 0.3 nm or below (Figure S1).

**Figure 2:**
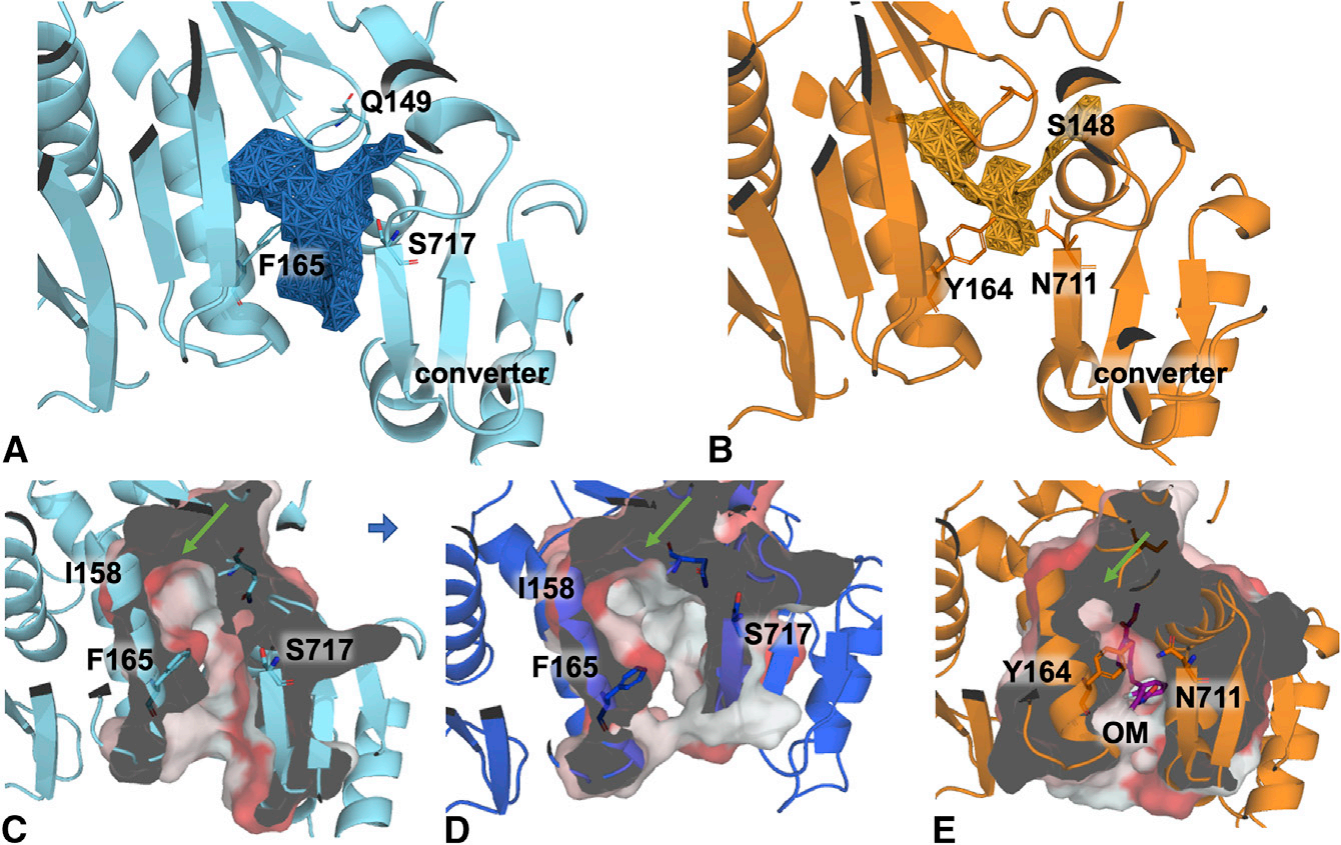
Comparison of the pocket shape in the two isoforms. (*A*/*B*) MDpocket frequency grids. Points that are found to be part of a pocket for at least 45% of the frames are shown as points connected by edges for the combined skeletal M1 and M2 (*A*) and the cardiac (*B*) MD simulations (OM-free). The three residues that differ in the two sequences are shown as sticks. (*C*/*D*/*E*) Surface representation of the pocket in the M1 (*C*) and M2 (*D*) models, and in OM-bound cardiac myosin (*E*). Representative structures from the most populated clusters observed in 300-ns MD simulations are used for the skeletal models, whereas the energy-minimized structure of the complex from Hashem et al. (7) is used for the cardiac isoform. The residues lining pockets identified by fpocket in the OM binding region are shown as surface in (*C* and *D*), whereas residues within 0.8 nm of OM are shown in (*E*). The surface is clipped to show the internal shape of the pocket. The hydrophobicity score is color mapped on the surface from white (low hydrophobicity) to red (high hydrophobicity) using the Eisenberg scale (21). The position of the subpocket that becomes accessible in the skeletal models is indicated with a green arrow. Residue I158_s_ is also labeled to further highlight the location of the subpocket. To see this figure in color, go online.

We then compared the structural and dynamical features of the binding region in the skeletal models with those observed in previously performed simulations of human cardiac myosin (OM-free, PPS state) (7). MDpocket (20) was used to detect binding pockets and track them during the simulations. For each isoform and model, a total of 12,000 PDB snapshots extracted from all the corresponding 300-ns trajectories were used for the calculations (see methods). Pocket frequency grids, which indicate the fraction of time a given point is found to be part of a pocket, were visualized and compared (Fig. 2 *A* and *B*). A single grid for the skeletal isoform was generated by merging the results from both models. Physico-chemical properties such as pocket volume and accessible surface area (ASA) were also calculated and monitored during the simulations. Important differences were observed in the shape and size of the pocket in the two isoforms. The cavity in both skeletal models is larger, with volume values higher by ∼ 0.19 (M1) and 0.28 (M2) nm^3^ and ASA values higher by ∼ 1.1 (M1) and 1.3 (M2) nm^2^ than in the cardiac isoform (Table 1). The pocket observed in the OM-free cardiac simulations, although located in the same region as the skeletal one, is smaller in the central part close to two residues that differ in the two sequences (Y164_c_ and N711_c_, Fig. 2 *B*). In the skeletal models, in addition to having shorter side chains, these residues are farther away from each other (Fig. 2 *A*).

**Table 1:** Average pocket volume and ASA in the OM binding region during the MD simulations

Property	M1	M2	Cardiac^a^
Volume (nm^3^)	1.127	1.220	0.939
ASA (nm^2^)	6.866	7.093	5.810

a Calculations for the cardiac isoform were performed using the region defined by the skeletal pocket frequency isosurface (see methods for details).

The skeletal cavity is larger than the cardiac one also when comparing the skeletal models to the OM-bound cardiac structure (Fig. 2 *C*–*E*). Representative structures extracted from M1 (Fig. 2 *C*) and M2 (Fig. 2 *D*) simulations using a cluster analysis clearly show a large subpocket in the upper region (green arrow). This subpocket is also present in the cardiac structure, but it is smaller in size and blocked from the rest of the cavity by the Y164_c_ and N711_c_ side chains. As a consequence, this part of the pocket is not accessed by OM when bound to cardiac myosin (Fig. 2 *E*). Analysis of the composition (Table S4) shows that the subpocket tends to be hydrophobic in nature, with residues Y118_s_, L121_s_, F122_s_, I158_s_, P673_s_, and F675_s_ (and V676_s_ only in M1) forming a hydrophobic patch. Interestingly, a comparison of our MYH2 models with the recently solved x-ray structure of the closely related fast type IIb myosin isoform MYH4 from rabbit (PDB: 6YSY (22)) shows a remarkable similarity throughout these structures. The MYH4 binding pocket (Fig. S2 *C*) is more similar in shape to the pocket in the MYH2 models (Fig. S2 *A* and *B*) than the cardiac one (Fig. S2 *D*), especially in the region of the hydrophobic subpocket (green arrow). This provides independent validation of our models, considering that the MYH4 structure was not used in the homology modeling.

### Molecular basis of OM selectivity

Docking studies were carried out to investigate the molecular basis of the preferential binding of OM to cardiac myosin. OM was docked to the two models of skeletal myosin, and the resulting binding affinity and poses were compared with those observed for cardiac myosin. Multiple structures extracted from the MD simulations were used for each model to take into account changes in the shape of the binding site due to the protein dynamics. To ensure that the data for the two isoforms were comparable, binding affinity to cardiac myosin was calculated by re-docking OM to multiple structures of cardiac myosin extracted from previously performed OM-bound MD simulations (7). A total of 16 skeletal and 17 cardiac structures were used. The same docking parameters and procedure were used for the two isoforms (see methods).

**Figure 3:**
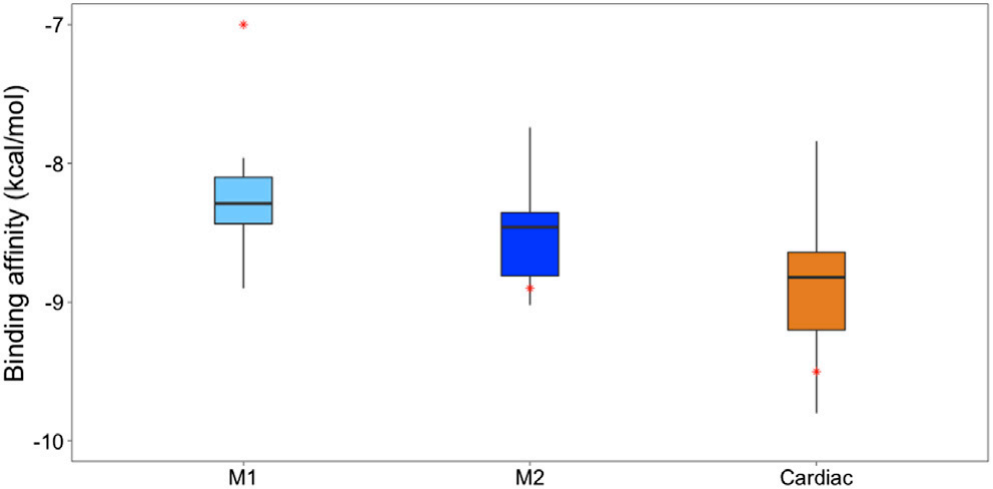
Comparison of predicted OM affinity to skeletal and cardiac myosin. The distributions of OM binding affinity values to structures extracted from MD simulations are represented as light blue (skeletal M1), blue (skeletal M2), and orange (cardiac) boxplots. Red stars represent the affinity values of OM to the initial energy-minimized M1 and M2 models for skeletal myosin, and to the x-ray structure (PDB: 5N69) for cardiac myosin. To see this figure in color, go online.

A comparison of the distribution of binding affinity values shows that binding is in general stronger to cardiac myosin compared with the skeletal models (Fig. 3), in agreement with the observed selectivity of OM (2). The cardiac distribution (orange) is shifted toward more negative values, reaching a minimum of −9.8 kcal/mol. The affinity of OM to around half of the cardiac myosin structures is below −9.0 kcal/mol, whereas skeletal affinities peak at around −8.2 kcal/mol with maximum values above −8 kcal/mol.

Interestingly, docking OM to the starting structures (which correspond to initial models after energy minimization) leads to very different values for M1 and M2 (red stars in Fig. 3 and Table S5). The M2 value is closer in affinity to the one observed for the cardiac x-ray structure (red star in the cardiac category), which reflects the higher similarity of the M2 model to the template (Table S3) and the higher similarity of the M2 OM binding pose to the x-ray one (Figure S3) compared with M1. When multiple structures from MD are considered for the docking, M1 and M2 values become much closer, and the two distributions almost completely overlap, indicating that during the simulations the two models converge toward a more similar behavior.

All myosin-OM complexes from the ensemble docking were clustered based on the volume overlap of the bound ligand to identify recurring binding poses. For each structure, poses from five different seeds were considered, leading to a total of 80 (5 × 16) and 85 (5 × 17) complexes for skeletal and cardiac myosin, respectively. A greater number of clusters were found for the skeletal complexes (Table S6) than the cardiac ones (Table S7), indicating a higher variability in the binding poses. A single dominant cluster was found for cardiac myosin (cluster 1 in Table S7), accounting for almost 30% of all the structures. The representative pose from this cluster is in very good agreement with the x-ray myosin-OM complex (Fig. S4 *B*), and its binding affinity is the second lowest value among the pose representatives. Skeletal binding poses were more uniformly spread over the different clusters, with no cluster having a population higher than 10%.

**Figure 4:**
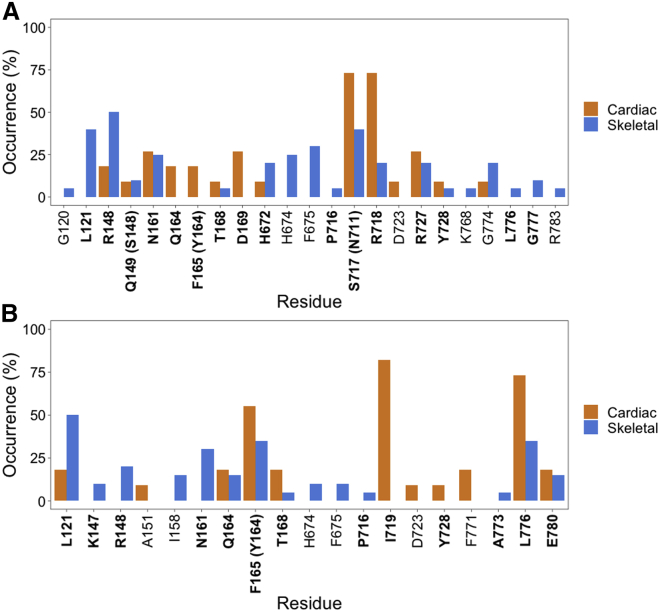
Comparison of OM-myosin interactions in the two isoforms. Occurrence of hydrogen-bonding (*A*) and hydrophobic (*B*) interactions identified by PLIP (23) in the skeletal (blue) and cardiac (orange) complexes. Occurrence is calculated across all the cluster representatives of the OM complexes for each isoform. Residues are named using the skeletal MYH2 sequence and numbering (cardiac MYH7 names and numbers are indicated in parentheses where the two sequences differ). Residues in the native OM binding region (listed in Fig. 1*C*) are shown in bold. To see this figure in color, go online.

A comparison of the hydrogen-bonding (Fig. 4 *A*) and hydrophobic (Fig. 4 *B*) interactions observed in the cluster representatives confirms that OM binding poses are much more consistent for cardiac (orange) than for skeletal (blue) myosin. The different skeletal poses involve a greater number of different residues, with the most recurring ones (Leu121_s_ for both types of interactions, and Arg148_s_ and Ser717_s_ for hydrogen bonding) being found in only 50% of the complexes or less. Some of these residues are not found in the native OM binding site (Fig. 1 *C*. In particular, Ile158_s_, His674_s_, and Phe675_s_ are part of the skeletal-specific hydrophobic subpocket described in the previous section, which is less accessible in the cardiac structure (Fig. 2). The cardiac binding poses show much less variability and more consistency, with fewer residues involved and higher occurrence values. Indeed, residues N711_c_ and R712_c_ form hydrogen-bonding interactions in 73% of the complexes, whereas Ile713_c_ and Leu770_c_ are involved in hydrophobic interactions in 73% of the cases or more.

**Figure 5:**
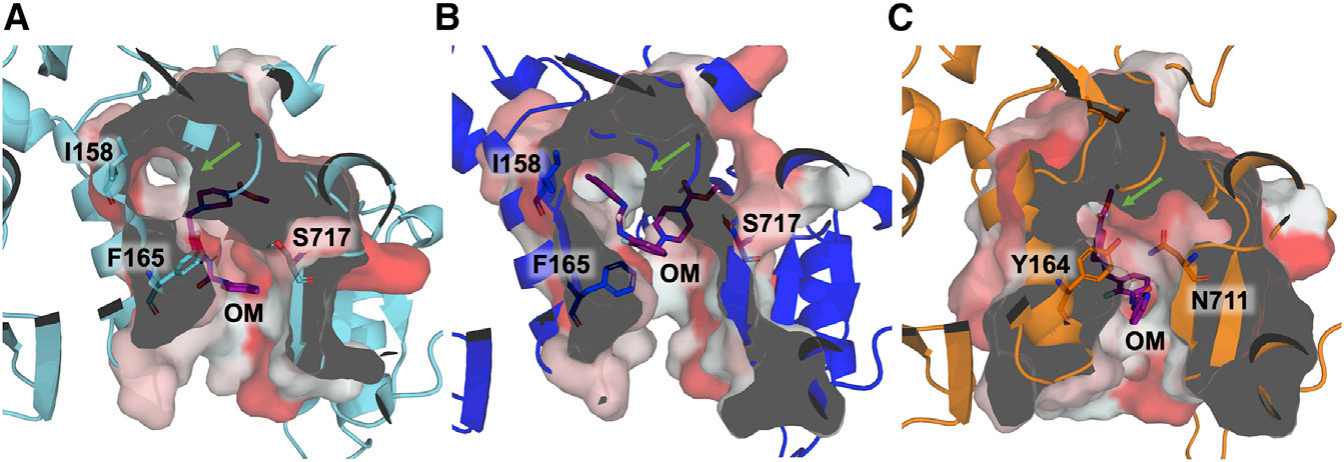
Comparison of OM binding modes in the two isoforms. OM-myosin complexes are represented to illustrate OM binding poses in (*A*) the cardiac-like OM-skeletal myosin complex (cluster 10 in Table S6), (*B*) the representative of the most populated OM-skeletal myosin cluster (cluster 17), and (*C*) the energy-minimized OM-cardiac myosin complex from Hashem et al. (7). The surface is clipped to show the internal shape of the pocket. The hydrophobicity score is color mapped on the surface from white (low hydrophobicity) to red (high hydrophobicity) using the Eisenberg scale (21). The position of the subpocket that becomes accessible in the skeletal models is indicated with a green arrow. To see this figure in color, go online.

Comparing the structures of the most populated binding poses shows that in the skeletal isoform the methylpyridine end of OM partially occupies the hydrophobic subpocket (Fig. 5 *B*), which is instead empty in the cardiac complex (Fig. 5 *C*). A “cardiac-like” pose was also found for the skeletal isoform, but this pose was only observed once, and its associated binding affinity (−8.3 kcal/mol) was still lower in comparison to OM affinity to cardiac myosin (−9.2 kcal/mol for the most populated pose or −9.5 kcal/mol for the x-ray structure).

**Figure 6:**
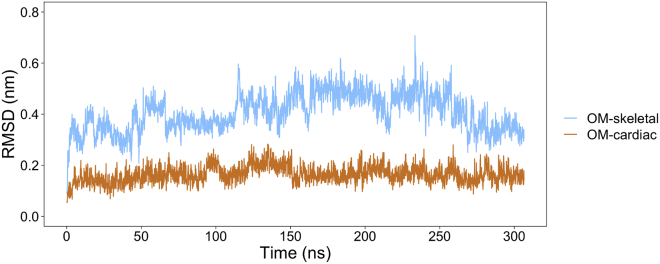
Comparison of OM binding stability in MD simulations of the two isoforms. The time evolution of OM RMSD (non-hydrogen atoms) from the starting structure is plotted for a representative MD simulation of OM-bound skeletal (cardiac-like binding pose M1_OM1, light blue) and cardiac (orange) myosin. RMSD values were calculated after fitting the system to the initial structure using the protein C-alpha atoms to take into account the roto-translational motions of the ligand inside the pocket. To see this figure in color, go online.

The stability of OM binding to skeletal myosin was further investigated using MD simulations. Poses with high (M1_OM1), intermediate (M1_OM2), and low (M2_OM3) similarity to the native cardiac one (Table S8) were selected to take into account the large variability of binding poses observed for the skeletal isoform. The pose stability during the simulations was monitored by calculating the RMSD of the position of nonhydrogen OM atoms with respect to their starting coordinates and compared with previous simulations of OM-bound cardiac myosin (7). RMSD values were always higher in skeletal simulations (Fig. S5) than the cardiac ones (Fig. S6), with averages around 0.38 nm (skeletal) and 0.17 nm (cardiac). A low stability was observed especially for the cardiac-like binding pose M1_OM1 (Fig. 6), which confirms that this pose is less favored in the skeletal isoform.

A comparison of the hydrogen-bonding interactions (Fig. S7 and Table S9) shows that a higher number of strong hydrogen bonds is formed in the cardiac binding pose compared with the skeletal ones. Indeed, although OM forms high occupancy (>50%) interactions with three cardiac residues (Asn711_c_, Asp168_c_, and Arg712_c_), the skeletal binding poses only have two (M1_OM1 and M2_OM3) or one (M1_OM2) high-occupancy hydrogen bonds (Table S9). Moreover, no single residue is involved in hydrogen bonding in all three skeletal complexes with an occupancy of over 10%, indicating a significant heterogeneity in the interactions of the three poses.

A different behavior was found when comparing the OM-myosin hydrophobic contacts for the two isoforms. A higher number of stable hydrophobic interactions were observed in the skeletal poses (bold in Table S10) compared with the cardiac one (Table S11). Only one stable contact (Phe165_s_) was observed in all the skeletal poses, confirming the diversity of interactions. Several contacts with the residues in the hydrophobic subpocket (highlighted in gray in Tables S10 and S11) were observed during the M1_OM2 and M2_OM3 simulations, whereas in the cardiac and cardiac-like skeletal pose M1_OM1, these were either absent or weak (Leu120_c_ and His492_c_).

## Discussion

In this work, we have studied the structure and dynamics of the OM binding site in cardiac and skeletal myosin to gain new insights on the molecular basis of OM selectivity and provide useful guidelines for the rational design of compounds that are selective toward skeletal isoforms.

Our approach included the generation of multiple structural models for skeletal myosin and their subsequent refinement via MD. Moreover, ensembles of structures extracted from MD simulations, rather than single structures, were used to compare the two myosin isoforms. Taking into account the effect of protein dynamics on the shape of the binding region was particularly important in this study because (1) for one of the models (M2), differences from the cardiac isoform were only evident after MD refinement, and (2) differences between the initial M1 and M2 skeletal models were reduced in the MD simulations, which consistently showed a larger binding site and weaker OM binding affinities for both skeletal models compared with the cardiac ensemble.

Although many of the amino acids forming the site are conserved in the two isoforms, differences at key positions were found to be responsible both for the larger skeletal pocket and for its decreased affinity for OM. The Tyr164_c_-Asn711_c_ pair of residues in the middle of the cardiac site is replaced by Phe165_s_-Ser717_s_ in the skeletal one. Due to shorter side chains and the absence of the phenolic OH group, these residues are not able to interact in the skeletal structures and are located farther from each other than in the cardiac one. This is accompanied by an increase in volume in the central region of the skeletal binding site and an increased accessibility of nearby regions that include a hydrophobic subpocket. This region is more buried than the native OM binding site, and in the OM-cardiac myosin complex, it is empty and separated from OM by the Tyr164_c_-Asn711_c_ interacting pair.

In agreement with experimental observations (2), OM was found to bind more strongly to the cardiac isoform compared with the skeletal one, as indicated by the relative order of binding affinity values, consistency of binding poses in ensemble docking calculations, and stability of interactions during MD simulations. The larger binding site in the skeletal myosin isoform led to a greater variability in OM binding poses in the ensemble docking calculations, and differently from the cardiac results, no dominant pose was identified. Even in the skeletal binding pose most similar to the native cardiac one, OM binds slightly deeper in the pocket as allowed by the larger site and by the missing interaction with Ser717_s_, whereas in the cardiac site the longer side chain of Asn711_c_ can reach up to the urea group of OM and keep it in its native cardiac position. For all the skeletal poses analyzed with MD simulations, the number of highly stable hydrogen bonds (occurrence of over >0.5) was lower than that observed in the native cardiac one. Consistent with this, higher RMSD values and fluctuations were observed for OM in all skeletal simulations compared with the cardiac ones, indicating a higher mobility of the ligand and thus an overall lower stability of binding.

It is to be noted that possible skeletal-specific OM-induced fit effects are not taken into account in the skeletal binding affinity values from docking. However, the MD simulations starting from the docked OM-skeletal myosin complexes (including the cardiac-like binding pose) show that even when allowing for at least partial adaptation of the protein structure to OM, the binding of OM is less stable than in the cardiac simulations.

When docked to skeletal myosin, OM explored regions near the native cardiac binding site, in particular the hydrophobic subpocket, which was partially occupied in the most recurring skeletal binding pose. The subpocket residue Leu121_s_ was found to be in contact with OM in different skeletal binding poses, whereas it seemed to contribute less to cardiac binding. In the skeletal poses analyzed by MD, the total number of residues in stable contact with OM was lower than for the cardiac simulations, indicating a less optimal shape complementarity. However, skeletal poses showed a higher number of stable hydrophobic contacts compared with the cardiac simulations, including contacts with the subpocket residues Leu121_s_, Pro673_s_, and Phe675_s_.

The differences between the cardiac and fast type IIa skeletal myosin isoforms presented in this work, in addition to providing a rationale for the selectivity of the cardiac modulator OM, also give a structural framework for the design of novel compounds that can selectively bind skeletal myosin. The larger skeletal binding site indicates that compounds could be designed so that they can bind to the skeletal isoform but not to the cardiac one. Indeed, compounds that can also target the hydrophobic subpocket could be expected to be selective for the skeletal isoform because of the reduced accessibility of this region in cardiac myosin. The availability of skeletal-selective myosin activators could have a great impact in the development of treatments for skeletal muscle diseases/myopathies where myosin function is disrupted directly (e.g., mutations in MYH2 (24) or in other sarcomeric proteins interacting with it (25,26)) or indirectly (e.g., sarcopenia (27)).

## Methods

### Homology modeling

In this work, two models of the motor domain of human MYH2 (UniProt: Q9UKX2) in the PPS state were generated using two different approaches in order to assess the dependence of the results from the starting structure.

The first model of the motor domain (M1, residues 1–789) was generated using the stand-alone program Modeller (28) (version 9.15). A BLAST (29) search of the PDB database identified the x-ray structure of bovine cardiac OM-bound myosin (PDB: 5N69 (8)) as the best PPS template on the basis of sequence identity to the target (79.8%), resolution (2.45 Å), and coverage (99%). The OM-bound structure was preferred to the Apo one (PDB: 5N6A (8)) not only for its superior resolution and coverage, but also because it features the primed lever arm conformation stabilized by OM (8). An additional x-ray structure of bovine cardiac myosin (PDB: 6FSA (30)) and an MD-refined structure (10) of human cardiac myosin (UniProt: P12883) were used as secondary templates to model three loops (209–217, 625–650, and 737–743), which are not solved in the main template. The alignment of the templates and target sequences was carried out with PRALINE (31). A total of 100 structures were generated by Modeller, and the one with the best DOPE score (32) was selected as the best model and used for further calculations. The second model (M2, residues 1–800) was generated with the “user template” mode of the web-based server SWISS-MODEL (33), which uses the target sequence and a user-specified template (PDB: 5N69) as input to produce a complete energy-minimized model. Low C-alpha RMSD values (Table S3) were observed between the templates and models, and between the models themselves. The M2 structure was found to be the most similar to the template, with an RMSD of only 0.011 nm.

The two models were further refined by energy-minimizing them with an all-atom force field and using explicit solvation (see Molecular dynamics simulation below). Quality assessment with QMEAN (34) and MolProbity (35,36) showed a better overall quality for the energy-minimized M2 model as measured by the overall MolProbity and QMEAN scores (Table S2).

### Molecular dynamics

#### System preparation and simulation protocol

MD simulations were carried out on the skeletal myosin models described in the previous section and compared with previously published simulations of cardiac myosin (7). Simulations were performed both on OM-free and OM-bound systems (see Table S12 for an overview). All systems were simulated with Mg^2+^, ADP, and Pi in the nucleotide binding site, with coordinates transferred from the x-ray structure 5N69 (8). Generation of parameters for OM and cofactors is described in Hashem et al. (7).

All skeletal myosin simulations were performed using GROMACS 2016 (37) and the Amber99SB^∗^-ILDN (38) force field. The protein was solvated with a truncated octahedral box of TIP3P (39) water molecules. Periodic boundary conditions were applied, and a minimum distance of 1.2 nm was set between the protein and the walls of the box. Ionizable residues were set to their standard protonation state at pH 7. Counterions were added to neutralize the system and reach an ionic strength of 50 mM. The total number of atoms in the systems was ∼186,500. The Particle Mesh Ewald method was used for electrostatic interactions, with a 0.9-nm cutoff for the direct space sums, a 0.12-nm FFT grid spacing, and a 4-order interpolation polynomial for the reciprocal space sums. A 0.9-nm cutoff was used for van der Waals interactions. Long-range corrections to the dispersion energy were included. The time step was set to 2 fs. Energy minimization, equilibration, and production simulations were run following the same protocol described in Hashem et al. (10). Production runs were initially performed for 100 ns and subsequently extended to 300 ns to check for convergence of results. Two OM-free replicas were run for each model, saving coordinates every 1 ps.

#### RMSD analysis

System stability was monitored by calculating the RMSD from the initial structure. For the whole protein analysis, long flexible loops, namely loops 1 (200–215) and 2 (625–650), and the cardiomyopathy loop (405–420), together with the excess C-terminus segment 785–795 for M2, were removed from the RMSD calculations to reduce the noise caused by their high flexibility. For the OM analysis, the RMSD was calculated for nonhydrogen OM atoms after the system was fitted to the initial structure using either OM non-hydrogen atoms or the protein C-alpha atoms to include roto-translational motions of the ligand.

#### Clustering of OM-free trajectories

A cluster analysis of OM-free 100-ns trajectories was performed using the gromos (40) method implemented in GROMACS, with an RMSD cutoff of 0.14 nm on structures sampled every 100 ps. For each model, the two replicas were concatenated before clustering. RMSD values were calculated on non-hydrogen atoms of the residues that correspond to those lining the OM binding site in cardiac myosin (Fig. 1 *C*).

Representatives of clusters with a population of over 10% were selected for docking studies (Table S13). Cluster centroids were used as representatives except for M2 cluster 1, where the centroid presented an uncharacteristically small binding pocket. In this case, the structure with the best compromise between pocket volume and similarity to the centroid of the cluster was selected.

Clustering was repeated after extension of the simulations to 300 ns, following the same procedure but using a slightly larger cutoff (0.17 nm) to account for the increased variability of structures. A high similarity (RMSD from 0.086 to 0.130 nm) was observed between the representative structures of the top-ranking clusters (population >10%) obtained for the 100-ns and 300-ns simulations, with the exception of the least populated (11.3%) M1 300-ns cluster, for which a 100-ns representative was found with RMSD <0.25 nm. Overall, this indicates that a good level of convergence is already met in the 100-ns simulations.

#### Analysis of the binding pocket with MDpocket

Dynamic changes of the target binding pocket throughout the MD simulations were evaluated with MDpocket (stand-alone version) (20). MDpocket is based on the repeated application of fpocket (41) on MD frames, which detects cavities as sets of α spheres. The frequency of occurrence of an α sphere is calculated at each point of a 0.1-nm grid centered on the protein and can be visualized as a frequency map.

For each set of OM-free skeletal myosin replicas (OM-free M1 and M2 in Table S12), MDpocket was run on the concatenated 300-ns trajectories sampled every 50 ps, and the grid points within the isosurface at a frequency of 0.45 were determined. For skeletal myosin, a single merged grid was created by selecting points that belong to at least one of the M1 or M2 grids. For cardiac myosin, MDpocket was run on four concatenated 300-ns trajectories of the OM-free PPS state (7). Default fpocket parameters for α sphere detection were used for all calculations.

To determine the residues forming the pocket in the skeletal myosin structures, fpocket was run on the representatives of top-ranking clusters from the 300-ns simulations. All the residues identified to be part of a pocket within the region encompassed by the skeletal isofrequency grid described above were selected (Table S4).

#### Analysis of OM-myosin interactions

The analysis of OM-myosin contacts was performed with the R package bio3D (42). A residue was considered to be in contact with OM if the minimum distance calculated over all pairs of non-hydrogen atoms was lower than 4 Å. Additionally, side-chain contacts were calculated by considering only nonhydrogen side-chain atoms. Occupancy of a given contact was calculated as the fraction of frames in which that contact was observed. High-occupancy contacts were identified as those with an occupancy of at least 0.5. Hydrophobic contacts were identified by analyzing the side-chain contacts for hydrophobic residues.

Hydrogen bonds were analyzed with the Hbonds plugin (43), using thresholds of 0.35 nm (donor-acceptor distance) and 30° (hydrogen-donor-acceptor angle).

#### Molecular docking

OM was docked to different skeletal and cardiac myosin structures using Autodock Vina (44). Autodock Tools (45) was used to prepare the ligand and protein structures for docking. Amide bonds in OM were set as rotatable (7). A similar protocol was used in our previous study (7) of OM binding to cardiac myosin, where Autodock Vina was shown to provide the correct relative order of OM affinity toward the PPS and the postrigor states.

Docking calculations were initially run on the cardiac x-ray structure (PDB: 5N69) to determine the docking parameters (exhaustiveness and number of runs). Six runs starting from different random seeds at exhaustiveness 2500 generated the same docking pose, which is in excellent agreement with the x-ray pose (Fig. S4 *A*). The binding affinity did not show any strong dependence on the seed used in the calculation, indicating a good level of convergence. The same parameters were used to dock OM to the initial energy-minimized M1 and M2 models of skeletal myosin.

OM was subsequently docked to a subset of structures extracted from MD simulations of each isoform to determine the dependence of the OM binding pose and affinity on the structure of the binding site. The skeletal myosin subset (16 structures in total) was composed of the representatives of the clusters with population >10% from both M1 and M2 trajectories (Table S13), together with top-ranking structures based on pocket volume. The cardiac myosin subset (17 structures in total) was similarly composed by the representatives of the most populated clusters extracted from OM-bound simulations, together with structures selected to represent the full range of OM binding affinity values as estimated from preliminary docking calculations (single runs with low exhaustiveness). The docking grid box was positioned and sized to include the residues that are part of the OM binding site (Fig. 1 *C*). Grid spacing was set to 0.1 nm, and the size of the box was adapted to the volume of the binding cavity. On average, this was 2.2 × 2.2 × 2.2 nm^3^. For each structure in the subsets, docking calculations were run with five different random seeds. For each run, the binding pose associated with the most negative binding affinity was selected for further analyses.

#### Clustering of docking poses

The OM docking poses generated as described above (80 for skeletal and 85 for cardiac myosin) were clustered with Maestro (Schrodinger suite, release 2021-2) to identify recurring binding modes. The poses were clustered on the basis of their volume score matrix (46) as calculated by Phase (Schrodinger suite, release 2021-2). For each pair of binding poses, volume scores were generated by dividing their volume overlap by the total volume occupied by OM in both poses. A hierarchical agglomerative clustering method (average linkage) was then applied using a merging distance of 0.52 for both sets of binding poses, which resulted in 20 and 11 clusters of skeletal and cardiac poses, respectively.

OM-myosin interactions were analyzed for all the cluster representatives using the Protein-Ligand Interaction Profiler (PLIP) web server (23) with default parameters.

The stability of selected OM binding poses to skeletal myosin was tested through MD simulations, which were performed following the protocol described above. Three cluster representatives were selected based on cluster population and their varying level of similarity to the native OM binding mode in cardiac myosin (Table S8).

## Author contributions

A.K.A. performed research and developed analysis tools; A.K.A. and A.F. analyzed data and wrote the manuscript; J.O. and A.F. designed and supervised research; J.O. reviewed the manuscript.

## Supporting material

Document S1. Figures S1–S7 and Tables S1–S13

Document S2. Article plus Supporting Material
