# Supplementary material for: Comparative study of binding pocket structure and dynamics in cardiac and skeletal myosin
Source: Biophys J. 2022 Nov 29;122(1):54–62. doi: 10.1016/j.bpj.2022.11.2942 (PMC9822794; doi:10.1016/j.bpj.2022.11.2942)
Supplement: Document S1. Figures S1–S7 and Tables S1–S13 [file mmc1.pdf]

Biophysical Journal, Volume *122*

Supplemental information

Comparative study of binding pocket structure and dynamics in cardiac and skeletal myosin

Anna Katarina Antonovic, Julien Ochala, and Arianna Fornili

Supplemental Information

Comparative study of binding pocket structure and dynamics in cardiac and skeletal myosin.

*Anna Katarina Antonovic[1], Julien Ochala[2, 3] and Arianna Fornili[1]**

1. School of Physical and Chemical Sciences, Queen Mary University of London, E1 4NS, London, United Kingdom
2. Department of Biomedical Sciences, University of Copenhagen, Blegdamsvej 3B, København N, DK-2200, Denmark
3. Centre of Human and Applied Physiological Sciences, King's College London, SE1 9RT, London, United Kingdom

Table S1. All residues mentioned in the text and their corresponding numbering in skeletal (MYH2) and cardiac (MYH7) myosin. Positions where the two sequences differ are highlighted in grey.

Skeletal myosin	Cardiac myosin
Asp90$_s$	Asp89$_c$
Met93$_s$	Met92$_c$
Tyr118$_s$	Tyr117$_c$
Gly120$_s$	Gly119$_c$
Leu121$_s$	Leu120$_c$
Phe122$_s$	Phe121$_c$
Cys123$_s$	Cys122$_c$
Tyr143$_s$	Tyr142$_c$
Lys146$_s$	Lys145$_c$
Lys147$_s$	Lys146$_c$
Arg148$_s$	Arg147$_c$
Gln149$_s$	Ser148$_c$
Ala151$_s$	Ala150$_c$
Ser157$_s$	Ser156$_c$
Ile158$_s$	Ile157$_c$
Asp160$_s$	Asp159$_c$
Asn161$_s$	Asn160$_c$
Gln164$_s$	Gln163$_c$
Phe165$_s$	Tyr164$_c$
Thr168$_s$	Thr167$_c$
Asp169$_s$	Asp168$_c$
Glu171$_s$	Glu170$_c$
Phe491$_s$	Phe488$_c$
His495$_s$	His492$_c$
Met496$_s$	Met493$_c$
Leu499$_s$	Leu496$_c$
Glu500$_s$	Glu497$_c$
Glu502$_s$	Glu499$_c$
Glu503$_s$	Glu500$_c$
Lys506$_s$	Lys503$_c$
Leu668$_s$	Leu662$_c$
Arg669$_s$	Arg663$_c$
Thr671$_s$	Thr665$_c$
His672$_s$	His666$_c$
Pro673$_s$	Pro667$_c$
His674$_s$	His668$_c$

Phe675$_s$	Phe669$_c$
Val676$_s$	Val670$_c$
Ile710$_s$	Ile704$_c$
Cys711$_s$	Cys705$_c$
Phe715$_s$	Phe709$_c$
Pro716$_s$	Pro710$_c$
Ser717$_s$	Asn711$_c$
Arg718$_s$	Arg712$_c$
Ile719$_s$	Ile713$_c$
Leu720$_s$	Leu714$_c$
Asp723	Asp717$_c$
Phe724$_s$	Phe718$_c$
Arg727$_s$	Arg721$_c$
Tyr728$_s$	Tyr722$_c$
Lys768$_s$	Lys762$_c$
Lys772$_s$	Lys766$_c$
Ala773$_s$	Ala767$_c$
Gly774$_s$	Gly768$_c$
Leu776$_s$	Leu770$_c$
Gly777$_s$	Gly771$_c$
Glu780$_s$	Glu774$_c$
Arg783$_s$	Arg777$_c$

Table S2. Model quality descriptors for energy-minimised structural models of MYH2.

		M1[c]	M2[d]
Protein Geometry	MolProbity score[a]	1.72	1.59
	Poor rotamers	31	23
	Favoured rotamers	548	570
	Ramachandran outliers	10	2
	Ramachandran favoured	736	743
Global Quality Scores	QMEAN[b]	-2.20	-1.74
	Cβ	-1.01	-0.21
	All atom	-0.12	0.84
	Solvation	0.03	0.23
	Torsion	-2.00	-1.78

[a]A lower MolProbity score indicates a higher model quality.
[b] For all five descriptors, a value close to zero indicates a quality comparable to the average quality of experimentally determined structures, whereas positive values indicate especially high, above average quality.
[c] Primary template: 5N69. Loop templates: 6FSA and MD. Modelling method: Modeller
[d] Primary template: 5N69. Modelling method: SWISS-MODEL

Table S3. Pairwise structural comparison of models (M1 and M2) and the main template (5N69). RMSD values (nm) calculated over C-alpha atoms are reported for each possible pair.

	**M1**	**M2**	**5N69**
**M1**		0.048	0.047
**M2**	0.048		0.011
**5N69**	0.047	0.011	

Table S4. Residues identified by fpocket as part of the binding region for M1 and M2 skeletal myosin MD simulations (OM-free). Fpocket was run on the representative structures of top-ranking clusters from the 300-ns M1 and M2 simulations. Residues in the hydrophobic sub-pocket are highlighted in grey.

M1	M2
Asp90$_s$	Asp90$_s$
-	Met93$_s$
Tyr118$_s$	Tyr118$_s$
Gly120$_s$	Gly120$_s$
Leu121$_s$	Leu121$_s$
Phe122$_s$	Phe122$_s$
Cys123$_s$	Cys123$_s$
-	Tyr143$_s$
-	Lys146$_s$
Lys147$_s$	Lys147$_s$
Arg148$_s$	Arg148$_s$
Gln149$_s$	Gln149$_s$
-	Ala151$_s$
Ser157$_s$	Ser157$_s$
Ile158$_s$	Ile158$_s$
-	Asp160$_s$
Asn161$_s$	Asn161$_s$
Gln164$_s$	Gln164$_s$
Phe165$_s$	Phe165$_s$
Thr168$_s$	Thr168$_s$
Asp169$_s$	Asp169$_s$
Glu171$_s$	Glu171$_s$
-	Phe491$_s$
His495$_s$	His495$_s$
Met496$_s$	-
Leu499$_s$	Leu499$_s$
Glu500$_s$	Glu500$_s$
-	Glu502$_s$
Glu503$_s$	Glu503$_s$
-	Lys506$_s$
-	Leu668$_s$
-	Arg669$_s$
-	Thr671$_s$
His672$_s$	His672$_s$
Pro673$_s$	Pro673$_s$

His674$_s$	His674$_s$
Phe675$_s$	Phe675$_s$
Val676$_s$	-
Ile710$_s$	-
Cys711$_s$	-
Phe715$_s$	-
Pro716$_s$	Pro716$_s$
Ser717$_s$	Ser717$_s$
Arg718$_s$	Arg718$_s$
Ile719$_s$	Ile719$_s$
Leu720$_s$	Leu720$_s$
Asp723$_s$	Asp723$_s$
-	Phe724$_s$
Arg727$_s$	Arg727$_s$
Tyr728$_s$	Tyr728$_s$
Lys768$_s$	Lys768$_s$
-	Lys772$_s$
Ala773$_s$	Ala773$_s$
Gly774$_s$	-
Leu776$_s$	Leu776$_s$
Gly777$_s$	Gly777$_s$
Glu780$_s$	Glu780$_s$
Arg783$_s$	-

Table S5. Binding affinity (kcal/mol) of OM to minimised skeletal models M1 and M2 and the 5N69 cardiac structure.

**Run**[a]	**M1**	**M2**	**5N69**
**1**	-7.0	-8.9	-9.5
**2**	-7.0	-8.9	-9.4
**3**	-6.9	-8.9	-9.5
**4**	-7.1	-8.9	-9.5
**5**	-7.0	-8.9	-9.4
Average	-7.0	-8.9	-9.5

[a] Different runs were performed with different seeds.

Table S6. Representative OM-skeletal myosin complexes and their binding affinity (kcal/mol) following clustering based on the volume overlap of the OM ligand.

Cluster number	Representative structure[a]	Binding affinity	Population
1	M1_rep1_run2	-7.9	4
2	M1_rep1_run4	-8.4	1
3	M1_rep2_run4	-8.4	5
4	M1_rep3_run4	-8.3	4
5	M1_rep3_run5	-8.2	1
6	M1_LP1_run0	-8.1	5
7	M1_LP1_run4	-8.4	5
8	M1_LP2_run1	-8.6	5
9	M1_LP3_run1	-8.0	4
10	M1_LP3_run3	-8.3	1
11	M1_LP4_run3	-8.9	5
12	M2_rep1_run5	-8.4	5
13	M2_rep2_run2	-8.9	5
14	M2_LP1_run1	-7.7	4
15	M2_LP1_run5	-7.7	1
16	M2_LP2_run2	-9.1	4
17	M2_LP3_run5	-8.4	8
18	M2_LP4_run2	-8.8	5
19	M2_LP5_run4	-8.3	3
20	M2_LP6_run5	-8.4	5

[a] Structures are labelled as M*x*_LP/rep*y*_run*z*, where M*x* indicates the model (M1 or M2) and run*z* the specific docking run. Structures selected on the basis of the pocket size are labelled as 'LP', while the cluster representatives are labelled as 'rep'.

Table S7. Representative OM-cardiac myosin complexes and their binding affinity (kcal/mol) following clustering based on the volume overlap of the OM ligand.

Cluster number	Representative structure[a]	Binding affinity	Population
1	rep1_run4	-9.2	25
2	rep4_run1	-8.5	10
3	rep5_run2	-8.7	7
4	BA5_run5	-9.1	5
5	BA11_run3	-9.8	5
6	BA14_run5	-9.2	8
7	BA16_run3	-8.8	10
8	BA18_run3	-9.1	5
9	BA26_run3	-7.8	5
10	BA29_run1	-8.7	1
11	BA29_run5	-8.7	4

[a] Structures selected on the basis of preliminary docking calculations are labelled as 'BA', while the cluster representatives are labelled as 'rep'.

Table S8. RMSD (nm) between representative OM-skeletal myosin binding poses and the native OM-cardiac myosin binding pose in the 5N69 X-ray structure. The RMSD value is calculated over all the OM heavy atoms after superimposing the protein structures.

Pose	Cluster number[a]	Representative Structure	RMSD
M1_OM1	10	M1_LP3_run3	0.47
M1_OM2	1	M1_rep1_run2	0.79
M2_OM3	17	M2_LP3_run5	0.95

[a] The same numbering is used as in Table S6.

Table S9. Frequency of occurrence of OM-myosin hydrogen bonds during 300-ns simulations of skeletal (M1_OM1, M1_OM2, M2_OM3) and cardiac myosin

Residue[a]	M1_OM1[b]	M1_OM2[b]	M2_OM3[b]	Cardiac[b,c]
$Arg148_s$	0.00	0.00	0.65	0.10
$Asn160_c$	0.00	0.00	0.00	0.05
$Gln163_c$	0.00	0.00	0.00	0.06
$Phe165_s$	0.00	0.00	0.11	0.08
$Asp169_s$	0.15	0.00	0.00	0.61
$His672_s$	0.00	0.16	0.52	0.00
$Asn711_c$	0.00	0.00	0.00	0.73
$Arg718_s$	0.61	0.80	0.00	0.53
$Arg727_s$	0.56	0.00	0.00	0.00
$Gly777_s$	0.20	0.00	0.00	0.00

[a] The cardiac numbering is used when the hydrogen bond was found only in the cardiac simulations

[b] Hydrogen bonds were calculated with VMD on trajectory snapshots sampled every 100 ps using a threshold of 3.5 Å on the Donor-Acceptor distance and 30° on the H-D-A angle. Only residues with frequency > 0.1 in at least one simulation are reported.

[c] Average occurrence calculated over all the replicas from Reference 7 in the main text.

Table S10. Frequency of occurrence of OM-myosin contacts in 300-ns MD simulations of skeletal myosin. High occurrence values (> 0.5) for hydrophobic side chains are highlighted in bold. Residues highlighted in grey are part of the hydrophobic sub-pocket.

Residue[a]	M1_OM1	M1_OM2	M2_OM3
$Phe165_s$	0.91 (**0.91**)	0.70 (**0.70**)	0.94 (**0.94**)
$Pro716_s$	0.78 (**0.59**)	0.80 (0.01)	0.95 (0.48)
$Ser717_s$	0.97 (0.94)	0.79 (0.67)	0.73 (0.73)
$Leu121_s$	0.29 (0.28)	0.99 (**0.98**)	0.99 (**0.89**)
$His672_s$	0.31 (0.31)	0.98 (0.98)	0.94 (0.94)
$Arg148_s$	0.28 (0.28)	0.82 (0.82)	1.00 (1.00)
$Arg718_s$	0.97 (0.59)	0.81 (0.81)	0.03 (0.03)
$Gly774_s$	0.87 (0.00)	0.02 (0.00)	0.87 (0.00)
$Gly120_s$	0.10 (0.00)	0.56 (0.00)	0.99 (0.00)
$Gln149_s$	0.50 (0.50)	0.04 (0.04)	0.85 (0.83)
$Leu776_s$	0.96 (**0.96**)	0.31 (0.31)	0.00 (0.00)
$Ala773_s$	0.38 (0.05)	0.01 (0.01)	0.88 (**0.84**)
$Tyr118_s$	0.00 (0.00)	0.13 (0.13)	0.87 (0.87)
$Pro673_s$	0.00 (0.00)	0.94 (**0.66**)	0.02 (0.00)
$His495_s$	0.00 (0.00)	0.96 (0.94)	0.00 (0.00)
$Phe122_s$	0.00 (0.00)	0.15 (0.03)	0.79 (0.00)
$Arg727_s$	0.92 (0.92)	0.01 (0.01)	0.00 (0.00)
$Ile719_s$	0.71 (**0.71**)	0.22 (0.22)	0.00 (0.00)
$Met496_s$	0.00 (0.00)	0.84 (**0.82**)	0.00 (0.00)
$Gly777_s$	0.76 (0.00)	0.00 (0.00)	0.00 (0.00)
$Lys772_s$	0.75 (0.00)	0.00 (0.00)	0.00 (0.00)
$Phe675_s$	0.00 (0.00)	0.75 (**0.75**)	0.00 (0.00)
$Phe771_s$	0.74 (0.49)	0.00 (0.00)	0.00 (0.00)
$Asp90_s$	0.05 (0.05)	0.00 (0.00)	0.67 (0.67)
$His674_s$	0.00 (0.00)	0.04 (0.04)	0.64 (0.64)
$Leu775_s$	0.65 (0.00)	0.00 (0.00)	0.00 (0.00)
$Glu780_s$	0.64 (0.64)	0.00 (0.00)	0.00 (0.00)
$Met93_s$	0.05 (0.05)	0.00 (0.00)	0.58 (**0.58**)
$Asp169_s$	0.56 (0.56)	0.04 (0.04)	0.00 (0.00)
$Phe492_s$	0.00 (0.00)	0.59 (**0.58**)	0.00 (0.00)
$Glu500_s$	0.03 (0.03)	0.52 (0.52)	0.00 (0.00)
$Asn161_s$	0.15 (0.15)	0.05 (0.05)	0.33 (0.33)
$Ile158_s$	0.00(0.00)	0.08 (0.08)	0.41 (0.41)
$Thr168_s$	0.44 (0.44)	0.00 (0.00)	0.00 (0.00)
$Tyr728_s$	0.31 (0.31)	0.00 (0.00)	0.00 (0.00)

$Cys123_s$	0.00 (0.00)	0.01 (0.01)	0.10 (0.05)

[a] A residue is considered in contact with OM if the minimum OM-residue distance calculated over non-hydrogen atoms is < 4 Å. Values in parentheses are calculated considering only the non-hydrogen atoms in the side chains. Only residues with frequency >= 0.1 in at least one simulation are reported.

Table S11. Frequency of occurrence of OM-myosin contacts in MD simulations of cardiac myosin. High occurrence values (> 0.5) with hydrophobic side chains are highlighted in bold. Residues highlighted in grey are part of the hydrophobic sub-pocket.

**Residue**[a]	**Average**[b]
$Tyr164_c$	1.00 (1.00)
$Arg712_c$	0.99 (0.95)
$Leu770_c$	0.95 **(0.94)**
$Asn711_c$	0.95 (0.92)
$Thr167_c$	0.94 (0.94)
$His666_c$	0.94 (0.94)
$Pro710_c$	0.90 **(**0.00**)**
$Asn160_c$	0.86 (0.86)
$Gln163_c$	0.83 (0.79)
$Ile713_c$	0.77 **(0.77)**
$Glu774_c$	0.76 (0.76)
$Tyr722_c$	0.72 (0.72)
$Arg147_c$	0.70 (0.69)
$Asp168_c$	0.64 (0.64)
$Lys146_c$	0.56 (0.55)
$Arg721_c$	0.50 (0.50)
$Gly771_c$	0.45 (0.00)
$Ser148_c$	0.42 (0.40)
$Leu120_c$	0.42 (0.41)
$Glu497_c$	0.26 (0.26)
$His492_c$	0.16 (0.16)
$Ala767_c$	0.15 (0.01)
$Glu500_c$	0.11 (0.11)

[a] A residue is considered in contact with OM if the minimum OM-residue distance calculated over non-hydrogen atoms is < 4 Å. Values in parentheses are calculated considering only the non-hydrogen atoms in the side chains. Only residues with frequency >= 0.1 in at least one replica are reported.

[b] Frequency values are taken from Reference 7 in the main text and averaged over all the replicas.

Table S12 Overview of MD simulations.

Myosin isoform	System	Length
**skeletal**	**OM-free M1 – replica 1**	300 ns
	**OM-free M1 – replica 2**	300 ns
	**OM-free M2 – replica 1**	300 ns
	**OM-free M2 – replica 2**	300 ns
	**OM-bound M1 (M1-OM1)**[a]	300 ns
	**OM-bound M1 (M1-OM2)**[a]	300 ns
	**OM-bound M2 (M2-OM3)**[a]	300 ns
**cardiac**	**OM-free (4 replicas)**[b]	4 x 300 ns
	**OM-bound (4 replicas)**[b]	4 x 300 ns

[a] OM-bound simulations were started from the representative binding poses listed in Table S8.

[b] Cardiac simulations were taken from Reference 7 and previously described there. The OM-free and OM-bound simulations were started from the humanized version of the X-ray structures of bovine cardiac myosin determined in the absence and in the presence of OM, respectively.

Table S13. Clustering analysis of OM-free skeletal myosin 100-ns trajectories.

	**M1**	**M2**
**Cluster #[a]**	**Population (%)**	**Population (%)**
1	39.8	36.1
2	19.3	30.0
3	17.7	-

[a] Only clusters with population > 10% are considered.

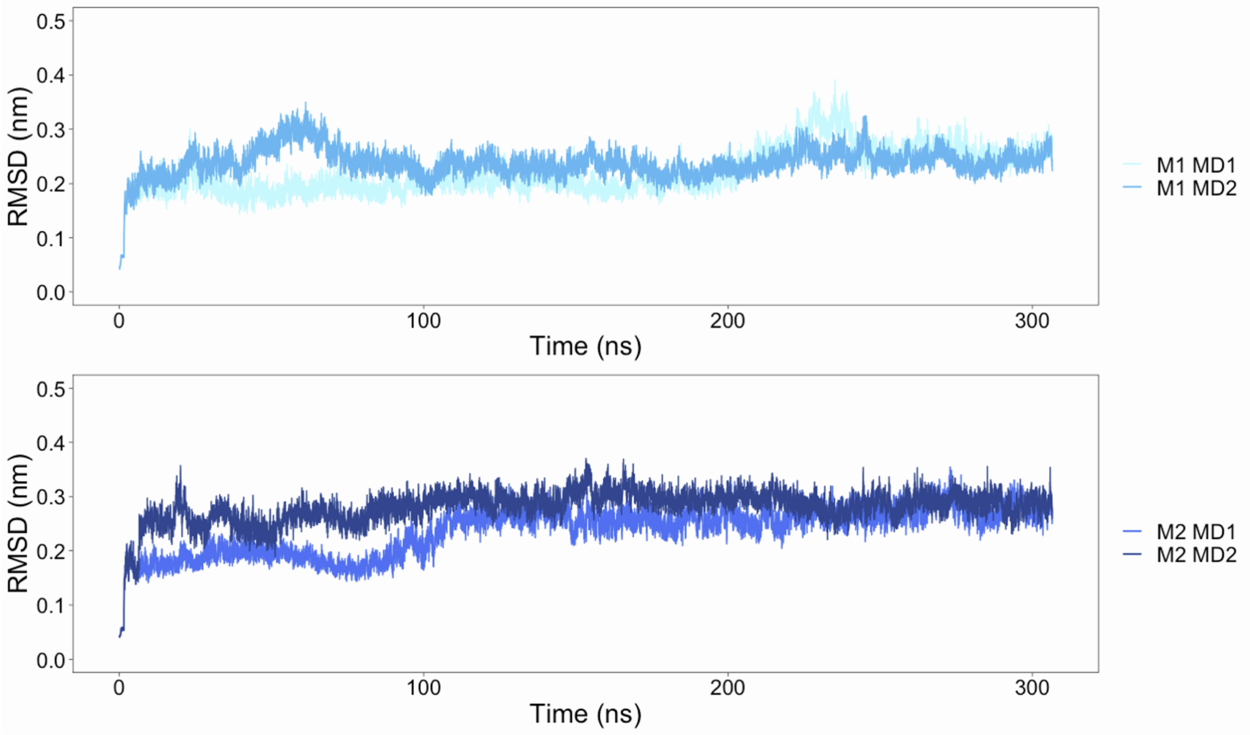

Figure S1. Time evolution of C-alpha RMSD values from the starting structure for skeletal M1 (light blue, top panel) and M2 (blue, bottom panel) simulations. Long flexible loops (loops 1 and 2, and the cardiomyopathy loop) were not considered in the calculation.

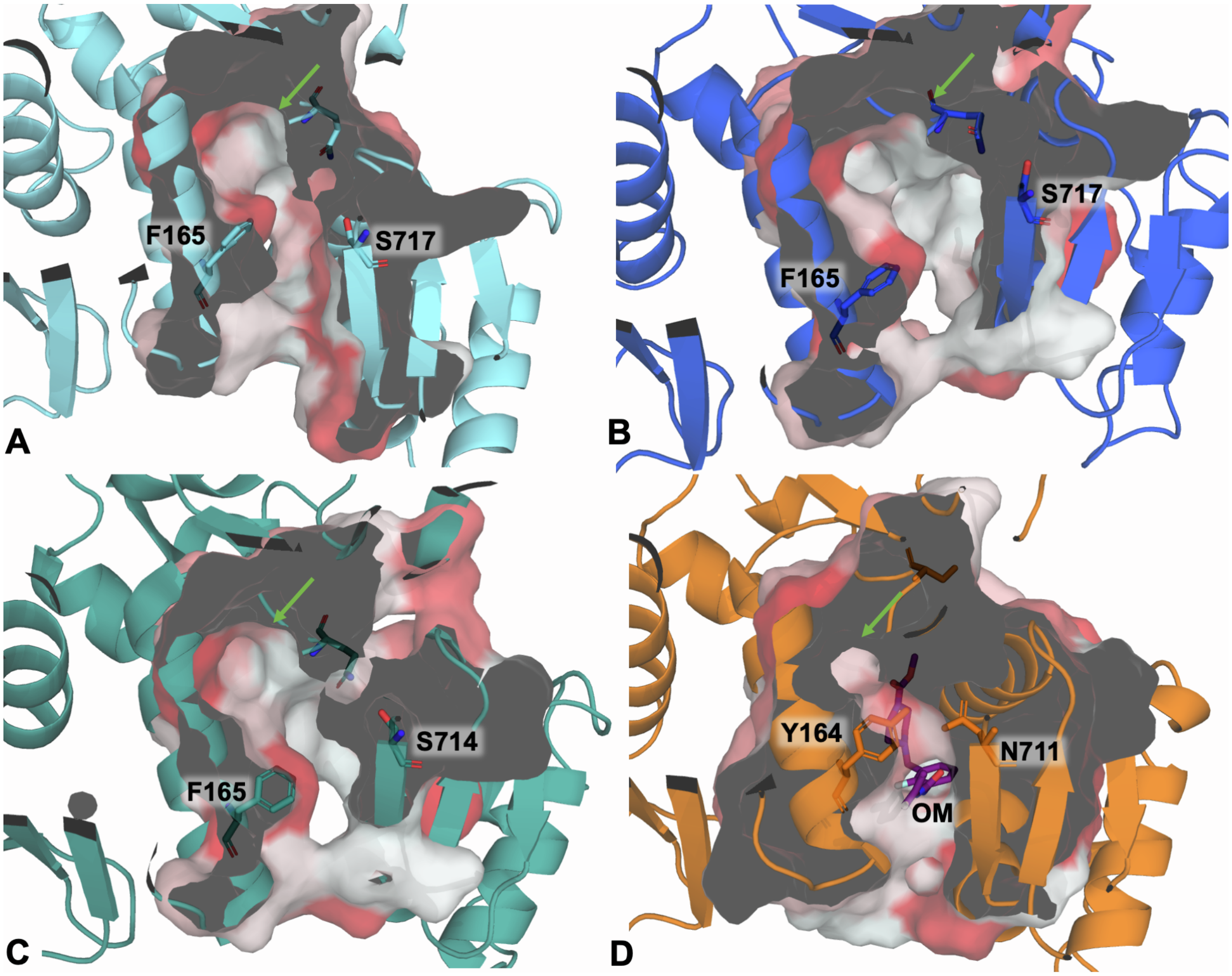

Figure S2. Comparison of pocket shape in different myosin isoforms. Surface representation of the pocket in M1 (**A**) and M2 (**B**) MYH2 models, in MYH4 (**C**) and in OM-bound MYH7 (cardiac) (**D**) myosin. Structures in **A**, **B** and **D** are described in the caption to Figure 2, while the MYH4 X-ray structure (PDB ID: 6YSY) was used for **C**, where the residues equivalent to those lining the MYH2 binding pocket (Table S4) are shown as surface. The position of the sub-pocket that becomes accessible in the skeletal isoforms is indicated with a green arrow.

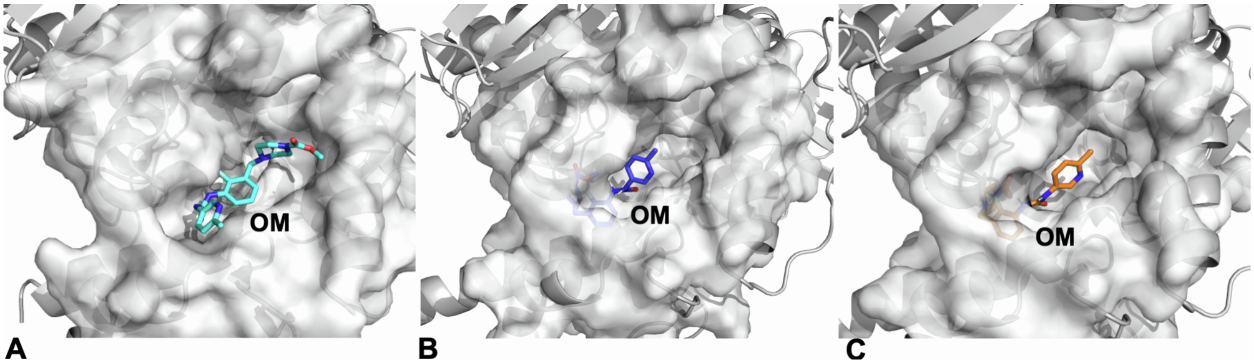

Figure S3. Stick representation of OM docked to the energy-minimised M1 (A) and M2 (B) models, with the residues lining the binding site represented as surface. The OM-cardiac myosin complex in the X-ray structure 5N69 is shown as a reference in panel C.

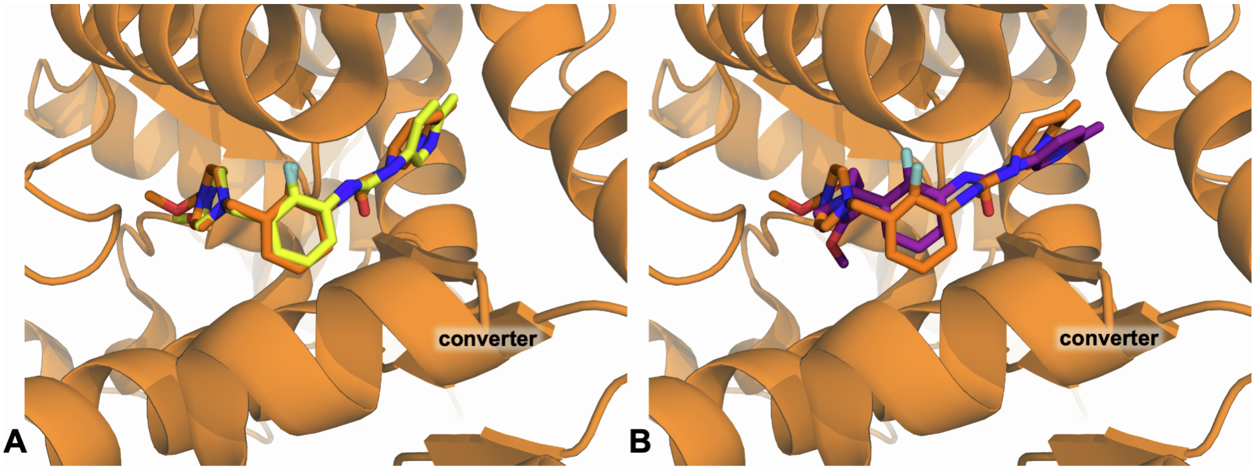

Figure S4. Superimposition between the X-ray structure of OM bound to cardiac myosin (PDB ID: 5N69, orange) and (A) OM re-docked to the cardiac X-ray structure (yellow) and (B) the most populated binding pose (cluster 1 in Table S7) observed when re-docking OM to representative structures of cardiac myosin from MD simulations (purple).

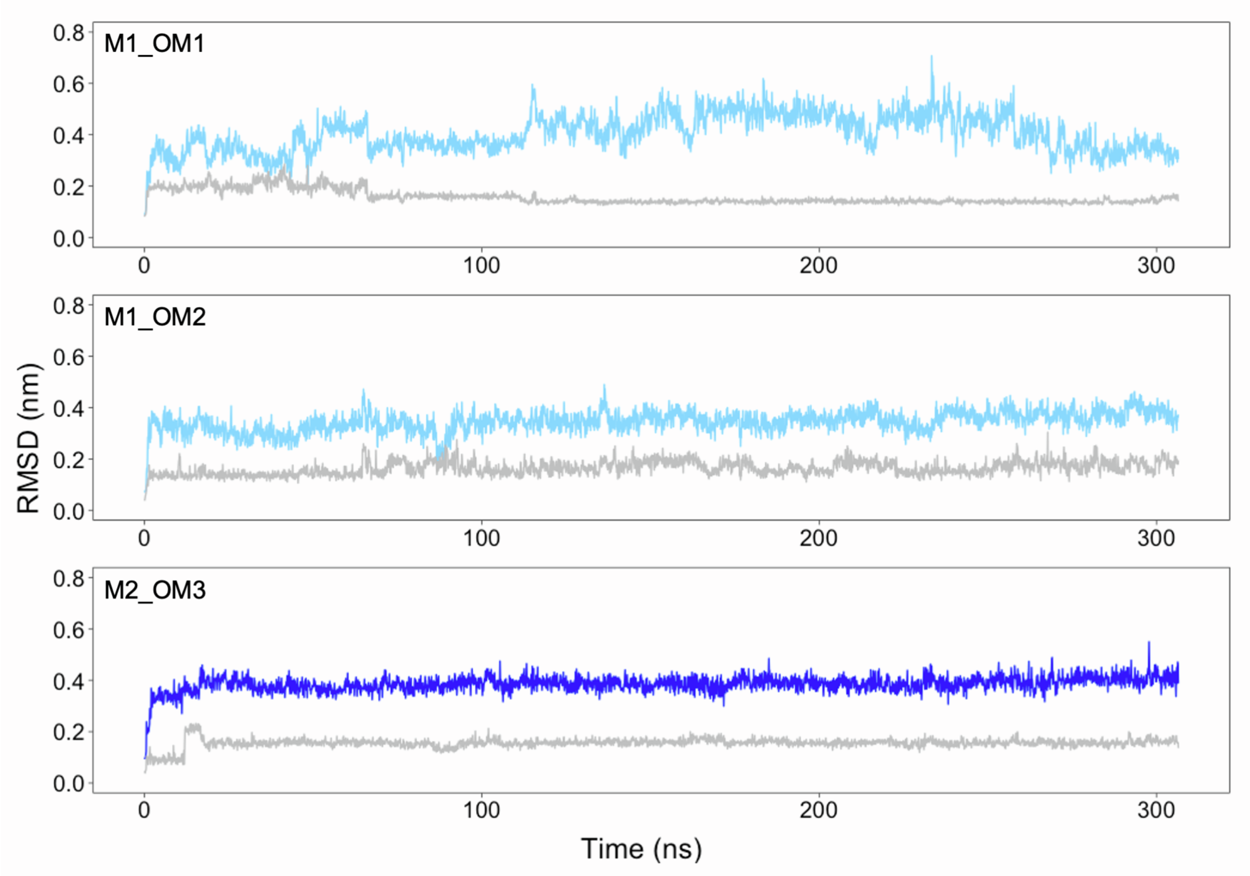

Figure S5. Time evolution of OM RMSD (non-hydrogen atoms) from the starting structure during MD simulations of skeletal myosin. RMSD values were calculated after fitting the system to the initial structure using either only OM non-hydrogen atoms (grey), to highlight changes in the internal OM conformation, or the protein C-alpha atoms (blue) to take into account also the roto-translational motion of the ligand.

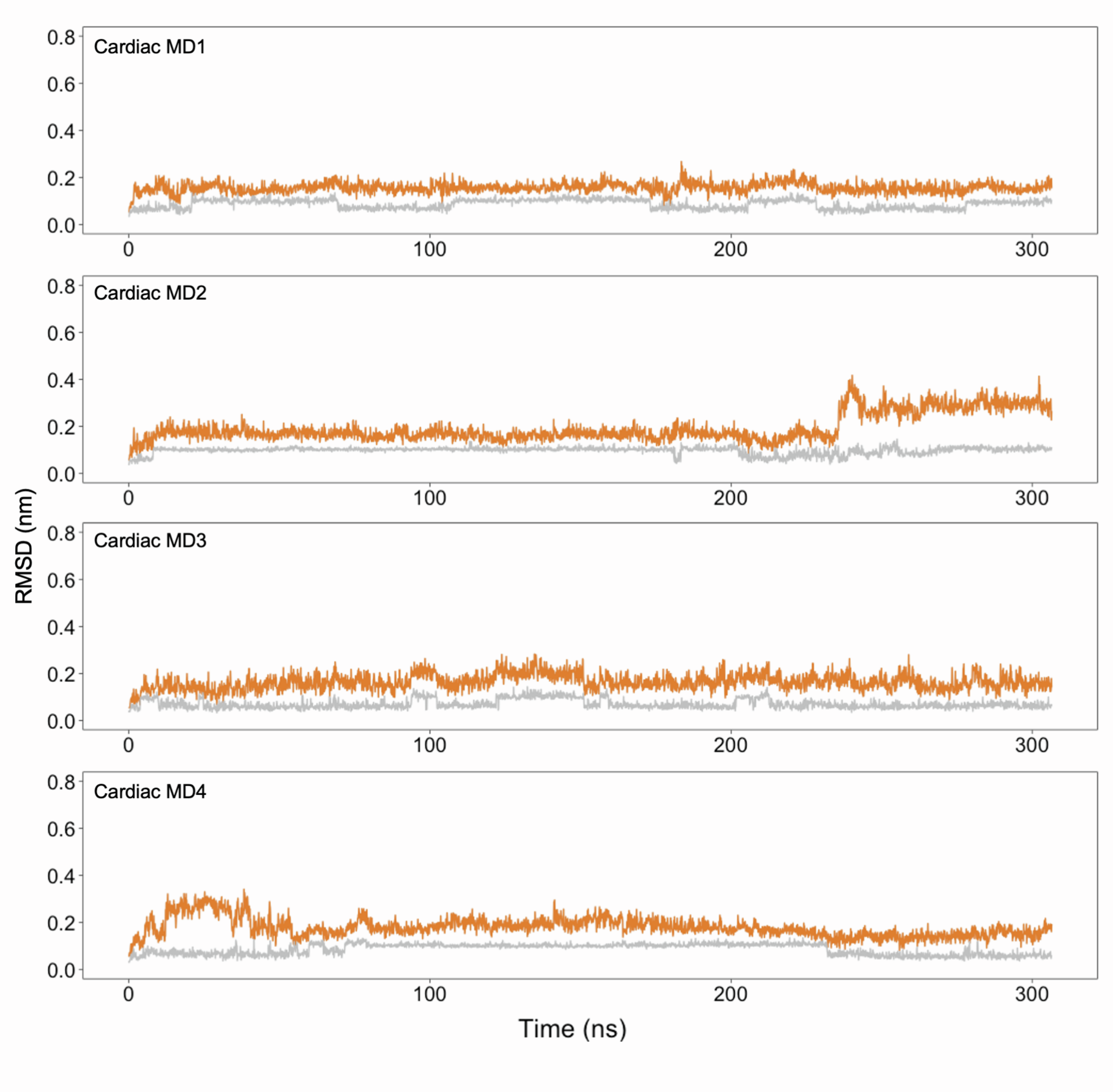

Figure S6. Time evolution of OM RMSD (non-hydrogen atoms) from the starting structure during MD simulations of cardiac myosin. RMSD values were calculated after fitting the system to the initial structure using either only OM non-hydrogen atoms (grey) to highlight changes in the internal OM conformation, or the protein C-alpha atoms (orange) to take into account also the roto-translational motion of the ligand.

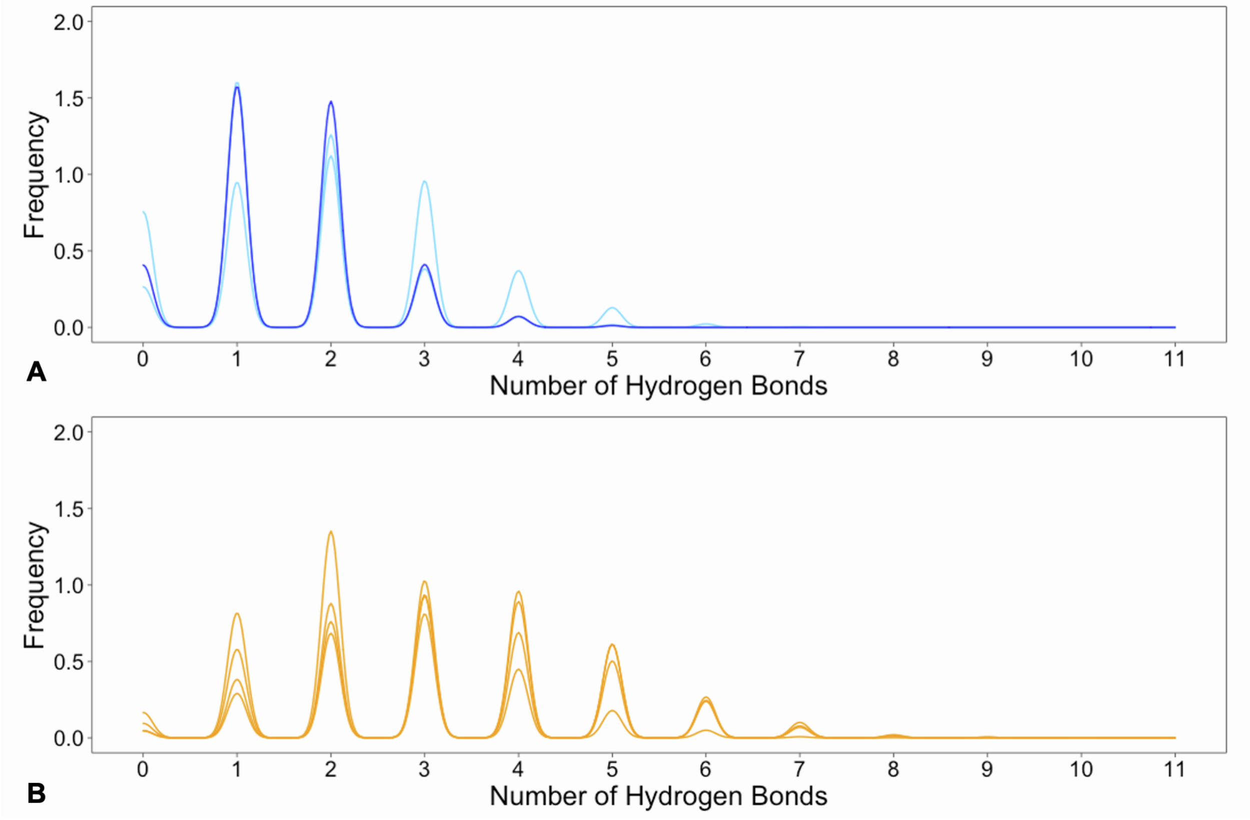

Figure S7. Frequency distribution of the number of OM-myosin hydrogen bonds formed throughout 300-ns MD simulations of skeletal (A) and cardiac (B) myosin.
